# Habitual nappers and non-nappers differ in circadian rhythms of *LIPE* expression in abdominal adipose tissue explants

**DOI:** 10.3389/fendo.2023.1166961

**Published:** 2023-06-09

**Authors:** Carolina Zambrano, Agné Kulyté, Juán Luján, Belén Rivero-Gutierrez, Fermín Sánchez de Medina, Olga Martínez-Augustin, Mikael Ryden, Frank A. J. L. Scheer, Marta Garaulet

**Affiliations:** ^1^ Department of Physiology, Regional Campus of International Excellence, University of Murcia, Murcia, Spain; ^2^ Biomedical Research Institute of Murcia, Instituto Murciano de Investigación Biosanitaria (IMIB)-Arrixaca-Universidad de Murcia (UMU), University Clinical Hospital, Murcia, Spain; ^3^ Endocrinology Unit, Department of Medicine Huddinge (H7), Karolinska Institutet, Karolinska University Hospital, Stockholm, Sweden; ^4^ General Surgery Service, Hospital Quirón salud, Murcia, Spain; ^5^ Department of Pathology, Stanford University School of Medicine, Stanford, CA, United States; ^6^ Department of Pharmacology, Centro de Investigación Biomédica en Red (CIBERed), Ibs Granada, Universidad de Granada, Granada, Spain; ^7^ Department of Biochemistry and Molecular Biology 2, Centro de Investigación Biomédica en Red, Enfermedades Hepáticas y Digestivas (CIBERehd), Ibs Granada, Instituto de Nutrición y Tecnología de los Alimentos (INYTA) José Mataix, Universidad de Granada, Granada, Spain; ^8^ Medical Chronobiology Program, Division of Sleep and Circadian Disorders, Brigham and Women’s Hospital, Boston, MA, United States; ^9^ Division of Sleep Medicine, Harvard Medical School, Boston, MA, United States; ^10^ Broad Institute of Massachusetts Institute of Technology (MIT) and Harvard, Cambridge, MA, United States

**Keywords:** lipolysis, obesity, circadian, *LIPE*, siesta

## Abstract

**Background and purpose:**

Napping is a widespread practice worldwide and has in recent years been linked to increased abdominal adiposity. Lipase E or *LIPE* encodes the protein hormone-sensitive lipase (HSL), an enzyme that plays an important role in lipid mobilization and exhibits a circadian expression rhythm in human adipose tissue. We hypothesized that habitual napping may impact the circadian expression pattern of *LIPE*, which in turn may attenuate lipid mobilization and induce abdominal fat accumulation.

**Methods:**

Abdominal adipose tissue explants from participants with obesity (n = 17) were cultured for a 24-h duration and analyzed every 4 h. Habitual nappers (n = 8) were selected to match non-nappers (n = 9) in age, sex, BMI, adiposity, and metabolic syndrome traits. Circadian *LIPE* expression rhythmicity was analyzed using the cosinor method.

**Results:**

Adipose tissue explants exhibited robust circadian rhythms in *LIPE* expression in non-nappers. In contrast, nappers had a flattened rhythm. *LIPE* amplitude was decreased in nappers as compared with non-nappers (71% lower). The decrease in amplitude among nappers was related to the frequency of napping (times per week) where a lower rhythm amplitude was associated with a higher napping frequency (r = -0.80; *P* = 0.018). Confirmatory analyses in the activity of *LIPE*’s protein (i.e., HSL) also showed a significant rhythm in non-nappers, whereas significance in the activity of HSL was lost among nappers.

**Conclusion:**

Our results suggest that nappers display dysregulated circadian *LIPE* expression as well as dysregulated circadian HSL activity, which may alter lipid mobilization and contribute to increased abdominal obesity in habitual nappers.

## 1 Introduction

Napping is a widespread practice worldwide and is considered a healthy habit in many countries (1, 2). However, several studies have linked napping with increased obesity and metabolic syndrome (MetS) risk (3–6). Therefore, the connection between napping and health is still a contradictory issue.

Our large-scale genome-wide association study (GWAS) in the UK Biobank (n = 452,633) and 23andMe (n = 541,333) identified 123 genome-wide genetic variants of napping (7). While research is needed to replicate these results in countries where sleep is a cultural habit (8), previous results based on Mendelian randomization analyses pointed to napping as a cause of abdominal obesity, the most harmful type of obesity (7).

Several mechanisms have been proposed to be involved in the effect of napping on abdominal obesity, most of them related to circadian disruption (9, 10). This relationship may be bidirectional. An impaired circadian system may result in nocturnal sleep disturbances and an increased need for sleep during the day (11, 12), resulting in napping. On the other hand, naps may cause poor nighttime sleep quality and lead to circadian disruption (13, 14). Regardless of the directionality, there is evidence that both sleep disruption and circadian disruption can increase the risk for obesity and have been found to impair glucose tolerance, reduce energy expenditure, and increase appetite and energy intake (15–17)—all considered obesogenic factors.

Many of these studies have focused on studying changes in the daily rhythms of the circadian hormone cortisol. Napping may induce elevations in post-nap cortisol levels (10), which, in turn, may increase fat deposition (18). While alterations in the circadian rhythms of genes directly involved in the lipogenic or lipolytic function of adipose tissue have been linked with obesity (19), the effect of napping on these factors has not been explored yet. We hypothesized that habitual napping is associated with a lower amplitude, a delayed acrophase, and a lower percent of rhythm (prominence of the rhythm) in the circadian pattern of the expression of *LIPE* (the major lipolytic gene in human adipose tissue), which may affect adipose tissue mobilization and induce abdominal fat accumulation.

Our group and others have shown that in human adipose tissue, there is a 24-h circadian rhythm in the activity of hormone-sensitive lipase (HSL), the primary enzyme responsible for releasing fatty acids from adipose tissue (20). A decrease in the daily rhythm amplitude of the activity of HSL has been related to a decrease in body fat mobilization (21). The expression of lipase E (*LIPE*), which encodes HSL, has also been shown to display 24-h circadian rhythms (20). We hypothesize that napping is associated with alterations in the circadian pattern of the expression of *LIPE*. Similar alterations may also be present in HSL activity. These changes in circadian rhythms may affect adipose tissue mobilization and induce abdominal fat accumulation.

To better understand the molecular mechanisms involved in the relationship between napping and abdominal obesity, we have studied potential differences in 24-h circadian *LIPE* expression between habitual nappers and non-nappers in abdominal adipose tissue biopsies obtained from participants with severe obesity who underwent laparoscopic gastric bypass surgery. Secondary analyses were also performed for HSL activity as a functional measure of mobilizing fat stores.

## 2 Materials and methods

### 2.1 Subject selection and classification

This cross-sectional study involved 17 participants with severe obesity (nine men and eight women; age (mean ± SD): 45.12 ± 11.47 years; BMI (mean ± SD): 42.05 ± 6.35 kg/m^2^; for further details, see **Table 1**). During the recruitment, nappers were selected to match non-nappers in age, obesity, and metabolic syndrome traits, to capture potential differences in the adipose tissue 24-h circadian rhythms of gene expression (*LIPE*) depending on napping *status* (and not because of age, obesity, or metabolic alterations).

**Table 1:** General characteristics of the participants, and habitual timing of sleep and meals in nappers and non-nappers.

	Total population (n = 17)		Napper (n = 8)		Non-napper (n = 9)		Student T test
	Mean	SD	Mean	SD	Mean	SD	^#^P-values
CHARACTERISTICS							
Sex (% female)	47.1		50		44.4		0.601
Age (years)	45.12	11.47	49.38	11.09	41.33	11.01	0.155
BMI (kg/m^2^)	42.05	6.35	39.12	7.26	44.66	4.30	0.071
Total body fat (%)	41.06	8.8	39.63	9.89	42.50	8.12	0.536
Metabolic syndrome traits							
Waist circumference (cm)	127.84	14.78	120.71	11.54	134.17	14.97	0.058
WHR	0.98	0.10	0.97	0.11	0.99	0.11	0.780
*Glucose (mmol/L)	6.49	1.90	7.16	2.33	5.90	1.28	0.180
*Triglycerides (mmol/L)	1.47	0.83	1.63	1.1	1.3	0.44	0.458
*HDL-c (mmol/L)	1.30	0.43	1.37	0.50	1.23	0.37	0.553
*Diastolic BP (mmHg)	82.35	6.09	83.6	7.78	81.22	4.26	0.435
*Systolic BP (mmHg)	137.52	15.32	137.7	11.19	137.33	18.97	0.958
MetS score	3.12	1.40	3.37	1.50	2.87	1.35	0.497
Timing of sleep							
*Napping characteristics*							
Nap onset (hh:mm)	–	–	15:37	0:30	–	–	–
Nap offset (hh:mm)	–	–	16:52	1:07	–	–	–
Nap frequency (times per week)	–	–	4.38	2.32	–	–	–
Nap duration (h)	–	–	1.18	0.70	–	–	–
*Nighttime sleep characteristics*							
Sleep onset (hh:mm)	0:30	1:06	0:27	1:24	0:33	0:50	0.878
Sleep offset (hh:mm)	7:47	0:58	07:35	0:59	7:58	0:58	0.445
Sleep duration (h)	7.27	1.37	7.12	1.56	7.41	1.25	0.676
Number of nocturnal awakenings/per night	0.47	0.87	0.88	1.12	0.11	0.33	0.070
Meal timing							
Breakfast onset (hh:mm)	8:55	1.01	8:34	0:39	9:14	1:13	0.198
Lunch onset (hh:mm)	14:41	0:36	14:48	0:42	14:34	0:31	0.468
Dinner onset (hh:mm)	21:42	0:49	21:42	0:41	21:41	0:59	0.955
Nighttime fasting duration (h)	10.87	1.43	10.46	1.01	11.23	1.71	0.283

BMI, Body mass index; WHR, waist-hip ratio**;** HDL-c, high density lipoprotein-cholesterol; BP, blood pressure; MetS, Metabolic syndrome. # Comparation between nappers and non-nappers; *Fasting conditions.

The participants underwent laparoscopic gastric bypass surgery at the General Surgery Service of the University Hospital Virgen de la Arrixaca (Murcia, Spain), and adipose tissue (AT) biopsies were obtained from abdominal subcutaneous AT at the end of the surgical procedure, which finished between approximately 12:00 and 13:00 h for all participants. Samples were collected from the upper left side of the abdomen in the subcutaneous area.

Protocols were reviewed and approved by the Institution Review Board of Virgen de la Arrixaca University Hospital, and written informed consent was provided by the participants before biopsies were obtained.

### 2.2 Anthropometric measures, body composition assessment, and metabolic syndrome traits

Body weight was determined in the participants wearing light clothes and bare-footed, using a digital electronic weighing scale. Height was determined using a Harpenden digital stadiometer (range 0.70–2.05 m) with the subject standing and their head in the Frankfurt plane. From these data, the BMI was calculated according to the following formula: weight/height^2^ (kg/m^2^). Total body fat (%) was measured by bio impedance with a Tanita Model TBF-300 (Tanita Corporation of America, Arlington Heights, IL, USA).

Waist and hip circumference were measured using a measuring tape, waist circumference was measured at the umbilicus level, and hip circumference was measured over the widest part of the great trochanter (22). The waist-to-hip ratio (WHR) was calculated as the ratio between waist and hip circumferences. Fasting serum concentrations of glucose, triglycerides, and HDL-c were determined with commercial kits (Roche Diagnostics, Mannheim, Germany). Arterial systolic and diastolic blood pressures were measured with a mercury sphygmomanometer while seated for at least 10 min. For each subject, a metabolic syndrome score (MetS score) was calculated, as the number of components of MetS, based on thresholds for waist circumference, fasting glucose (≥5.6 mmol/dl), triglycerides (1.7 mmol/dl), HDL-c (< 1.03 mmol/l for men, < 1.29 mmol/l for women), and systolic (≥ 130 mmHg) or diastolic blood pressure (≥ 85 mmHg) with a maximum value of 6 points (23).

### 2.3 Napping characteristics

Several questions about napping (known as “siesta” in this Spanish cohort) were asked such as “do you habitually take siesta?” (yes/no); if the answer is yes, then the timing (siesta onset and offset), frequency (times *per* week), and duration (minutes) of napping were asked. Those subjects who answered no siesta were considered non-nappers, whereas those taking siesta at least once *per* week were classified as habitual nappers.

### 2.4 Nighttime sleep characteristics

We estimated nighttime sleep onset and offset, as well as sleep duration, using the following questions: 1) “at what time do you go to sleep?” and 2) “at what time do you usually awake in the morning?” Sleep duration was determined as the difference between estimated sleep onset and offset. In addition, the following question was asked regarding the number of awakenings during nocturnal sleep: “how many times do you usually wake up during the night?”

### 2.5 Timing of meals

Habitual timing of food intake was also reported with the question “at what time do you usually have breakfast (lunch or dinner)?” and responses were in 30-min increments. In addition, nighttime fasting duration was calculated by the following formula: the timing of ending the last meal of the previous night (dinner offset) *minus the* timing of starting the first meal on the next day (breakfast onset).

### 2.6 Adipose tissue culture and adipocyte size measurements

After surgery, the adipose tissue biopsies (30–40 g of AT per patient) were cut into small pieces of 1–2 mm^3^ to promote better contact of adipose tissue with the culture medium, and the fragments were combined in each well to obtain an approximate weight of 1.5 g for each circadian time point (without technical replicates). Explants were cultured in a total volume of the *medium* of 2.5 ml (Dulbecco’s modified Eagle’s *medium* (DMEM)) that was supplemented with 10% fetal bovine serum (GIBCO), glucose (4.5 g/l), and a mixture of penicillin–streptomycin–glutamine (PSG from GIBCO #10378-016). In the laboratory, cultures were kept at 37°C for approximately 24 h in a modified atmosphere of 7% CO_2_. The next day, the old *medium* was replaced by DMEM *medium* with 1 g/l glucose supplemented with penicillin–streptomycin–glutamine (GIBCO PSG #10378-016) (without fetal bovine serum). Abdominal adipose tissue explants from participants (n=18) were cultured for a 24-h period and collected every 4 h for further analyses of gene expression. AT subcutaneous explants were distributed in six wells, one for each time point of the circadian cycle (i.e., Circadian Time zero (CT0 [8:00 h]) CT4 [12:00 h], CT8 [16:00 h], CT12 [20:00 h], CT16 [00:00 h], and CT20 [04:00 h]). Therefore, the first explant was collected at 8:00 am in a cryotube and frozen at -80°C, corresponding to Circadian Time zero (CT0). This time was selected for being the average habitual waketime of the subjects studied, and the timing when the *medium* was changed. Every 4 h, progressively, the other AT explants were collected in cryotubes and frozen in the same conditions for subsequent assessment of *LIPE* gene expression and HSL enzyme activity, obtaining the other five subsequent explants.

The remaining AT fragments not used for AT culture procedure were carefully frozen at -80°C. Later, we used them for subsequent measurements of the size of the adipocytes. Adipocyte diameter measurements were performed according to Sjöström et al. (24). The slide measurements were performed repeatedly and in different slices on the same AT sample, previously prepared and cut with a cryostat (LEICA Biosystem CM3050 S) to examine the variability of the measurements. The technical and operator reliability of these assessments and the variability (correlation factors) were originally examined by our group from duplicate measures in several participants (n = 8)— in the same slice (r = 0.99), in two different slices from the same AT sample (r = 0.99), and in slices observed by different operators (r = 0.94) (25).

### 2.7 RNA extraction: *LIPE* expression

RNeasy Lipid Tissue Kit (Qiagen, Hilden, Germany) was used according to the manufacturer’s recommendations for RNA extraction from a piece of 100 mg of each one of the six frozen explants. Quality and concentration were evaluated with the NanoDrop One spectrophotometer (Thermo Scientific). Subsequently, 50 ng of RNA was transcribed into cDNA using the iScript cDNA synthesis kit (Qiagen) and random hexamer primers (Invitrogen, Carlsbad, CA). PCR conditions and primers for the detection of *LIPE* mRNA and (18s rRNA, housekeeping gene) have been described previously (26).

According to the manufacturer’s guidelines, data were obtained as Ct values and used to calculate ΔCt values (ΔCt = Ct of the target gene-Ct of the housekeeping gene (18S) of each sample) (27). The relative changes in gene expression were calculated by the 2−ΔΔCt method (28). Afterward, in each subject’s data, normalization was performed using the median of the six-CT- point gene expression (2−ΔΔCt) of each subject. This approach has been previously shown to be an appropriate method for analysis of gene expression profiling, better than others such as total RNA globalization, centralization, or percentile normalization (29). Furthermore, the data were introduced in the software for cosine analyses following the guidelines of RITME^®^, version 4 (developed by A. Diez-Noguera, Univ. Barcelona, 2012).

### 2.8 *LIPE* circadian rhythmicity

To assess circadian rhythmicity, cosinor analysis was performed; for this purpose, normalized gene expression data were fit by 24-h sinusoidal waveforms, and we further defined the characteristics of the rhythms such as the timing of the peak (acrophase); the difference in the gene expression level between the maximum (or minimum) value of the cosine function and mesor (the amplitude); the mean value of *LIPE* rhythm fitted to a cosine function (mesor); and the percentage of the variance of the data explained by the cosine model or percent of rhythm (the prominence), which represents the strength and endurance of a rhythm. As secondary analyses, the activity of HSL was measured by Western blot as the ratio between phosphorylated HSL (pHSL) and total HSL (tHSL), using tHSL as loading control as described previously (**Supplementary Figure 1**) (20, 30).

### 2.9 Statistical analyses

The quantitative data of **Table 1** and **Supplemental Table 1** (31) were expressed as mean and standard deviation, and the differences between nappers’ and non-nappers’ characteristics were assessed by the Student T test. The qualitative variables (as sex) were presented as frequency and percentages, and differences between nappers and non-nappers were assessed by the chi-square test. The descriptive analyses were carried out by using IBM SPSS Statistics for Windows (version 28.0; Armonk, NY, USA).

A computer program was used for the cosinor analysis (RITME^®^, version 4, developed by A. Diez-Noguera, Univ. Barcelona, 2012), and to determine the different characteristics of 24-h circadian rhythms of *LIPE* expression: mesor, amplitude, acrophase, and percent rhythm or prominence. The percent of rhythm was calculated as the percentage of the variance accounted for by the fitted cosine model, which corresponds to the coefficient of determination R^2^ in regression analysis (32). The P value calculated to assess whether a rhythm was significant or not was obtained by dividing the data variance explained by the cosine model by the residual (or unexplained) variance, which gave us the F Snedecor coefficient. For the global population analyses, nappers’ and non-nappers’ rhythmicity was considered when the *P* value was <0.05, whereas for each individual, rhythmicity was considered when the percent of rhythm was > 60%, as previously reported (33, 34). ANCOVA analyses were used to determine differences in these characteristics between nappers and non-nappers, using sex and age as covariates. Further sensitivity analyses included night sleep duration as a covariate. The same analyses were performed for HSL activity.

The software CirWave^®^ (version 14.0, developed by R.A. Hut; available from https://www.euclock.org) was used as an extension of the cosinor analyses to define the cosinor curve, and the curve was drawn with GraphPad^®^ (version 8.0.2, GraphPad Prism^®^ from http://www.graphpad.com).

The level of significance for all statistical tests and hypotheses was set with a two-tailed P-value < 0.05 as statistically significant.

## 3 Results

The global characteristics of the studied population are summarized in **Table 1**. As expected by design, no significant differences were found in age, obesity, and MetS traits between habitual nappers and non-nappers. Every habitual napper had a midday postprandial nap (i.e., siesta) with napping onset after 15:00 h (15:37 h average). Siesta duration ranged from 30 to 120 min (2 h) with a mean duration of 1.18 ± 0.70 h. Within habitual nappers, three subjects were short nappers (siesta of ≤30 min), whereas five were long nappers (>30 min). No significant differences were found in the timing, duration, and number of nocturnal awakenings of nighttime sleep between habitual nappers and non-nappers. The timing of meals was also similar between both groups (**Table 1**).

**Figure 1:**
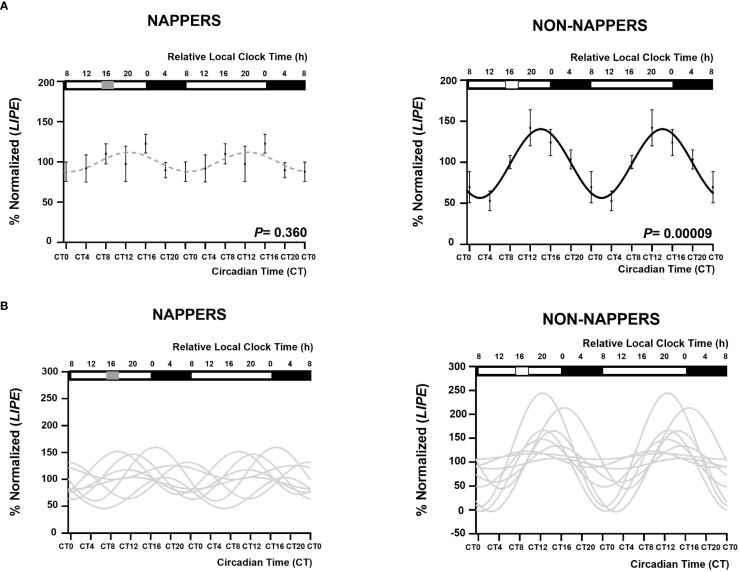
Rhythm expression of *LIPE* in subcutaneous abdominal adipose tissue explants in participants with severe obesity according to their habit of napping (n = 8; *P* = 0.360) or not napping. (n = 9; *P* = 0.00009). The global population analyses in both subgroups are represented in **(A)** represented by a black waveform sinusoidal curve if a significant cosine rhythm is present (in non-napper) and by a gray dashed line when there is no significance (nappers). Data (%normalized) are represented by black dots and the standard error of means as vertical lines. **(B)** represents each individual’s waveform. Among non-nappers, five of nine individuals displayed a significant rhythm in *LIPE* expression, whereas among nappers, only one individual (from eight) displayed a significant rhythm. The X-axes are represented both in relative local clock time (h; top X-axes) and in circadian time (CT; relative to the average habitual waking time and the *ex vivo* medium change; bottom X-axes). In the top X- axis, sleep timing is represented by a black rectangle and napping time is represented by a grey rectangle in napping and white square in non-napping individuals. In the four panels, data are double plotted to aid visualization of rhythmicity. The group average-based fit amplitude was 27 (% normalized *LIPE*), that of the non-nappers group was of 42 (% normalized *LIPE*), and that of the napper group was 12.

**Figure 2:**
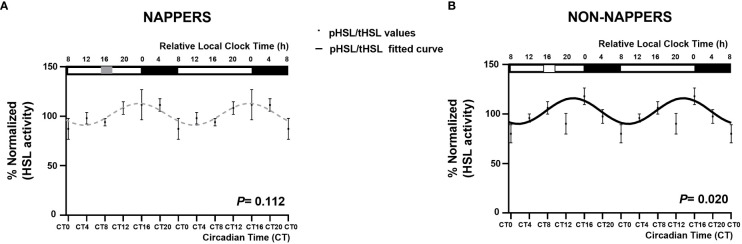
Rhythm expression of HSL activity in subcutaneous abdominal adipose tissue explants in participants with severe obesity according to their habit of napping (n = 8; *P* = 0.112) or not napping (n = 9; *P* = 0.020). The global population analyses in both groups are represented in non-nappers as a black waveform sinusoidal curve **(B)** and in nappers as gray dashed lines **(A)**. Data (%normalized) are represented by black dots and the standard error of means as vertical lines. The X-axes are represented both in relative local clock time (h; top X-axes) and in circadian time (CT; relative to the average habitual waking time and the *ex vivo* medium change; bottom X-axes). In the top X-axes, sleep timing is represented by a black rectangle, and napping time is represented by a gray rectangle in napping and a white rectangle in non-nappers.

**Figure 1** displays 24-h circadian rhythms of *LIPE* in *in vitro* subcutaneous abdominal adipose tissue explants in habitual nappers and non-nappers. In **Figure 1A**, global population analyses in both subgroups are represented, whereas **Figure 1B** represents each individual’s waveform. Globally, we found that adipose tissue explants from non-nappers exhibited robust circadian rhythms in *LIPE* expression (*P* = 0.00009) (**Figure 1A**). In contrast, nappers had a flattened rhythm (*P* = 0.360) (**Figure 1A**). Results were confirmed in HSL activity where we found significant circadian rhythms in non-nappers (*P* = 0.020), whereas significance was lost among nappers (*P* = 0.112) **Figure 2**.

**Table 2** shows the characteristics of the 24-h circadian rhythms of *LIPE* expression and the adipocyte diameter in nappers and non-nappers. Significant differences were found in amplitude, acrophase, and percent of rhythm between nappers and non-nappers (i.e., when comparing characteristics of the rhythm from each individual) toward a higher amplitude, advanced acrophase, and higher percent of rhythm in non-nappers (*P* < 0.05); similar results were found in HSL activity, although significance was not reached (**Supplementary Table 2**) (35). The 24-h average expression of *LIPE* was similar between both groups (*P* = 0.616) (after adjusting by sex, age, and nighttime sleep duration). Concerning adipocyte diameter, no significant differences were found between nappers and non-nappers (*P* = 0.792).

**Table 2:** Characteristics of the 24-h circadian rhythms of *LIPE* expression and adipose tissue in nappers and non-nappers.

	Total population (n = 17)		Napper (n = 8)		Non-napper (n = 9)		ANCOVA	ANCOVA
	Mean	SD	Mean	SD	Mean	SD	P-values*	P-values**
Rhythm characteristics *LIPE* expression								
Average (fold change or ΔCt)	12.75	1.51	13.04	1.52	12.50	1.55	0.691	0.616
Amplitude (% normalized)	43	32	30	14	55	40	0.062	0.035
Acrophase (hh:mm)	22:12	3:52	23:06	5:12	21:25	2:12	0.053	0.018
Percent of rhythm	44.54	28.66	35.18	20.71	52.86	33.19	0.159	0.038
ADIPOSE TISSUE								
Adipocyte diameter (µm)	97.61	8.72	96.43	7.1	98.80	10.77	0.537	0.792

µm, micrometers. *Adjusted for sex and age. **Adjusted for sex, age, and sleep duration night. Data were obtained from the median of each individual’s rhythm characteristics.

We performed secondary analyses within the napping group to better understand the characteristics of the 24-h circadian rhythms of *LIPE* expression in this group and the potential association with siesta traits and the timing and duration of nighttime sleep and meals. The results of these analyses showed that the amplitude of the *LIPE* rhythms was significantly correlated with the frequency of siesta toward a decreased amplitude with higher frequency of siesta per week (r = --0.80; *P* = 0.018). In addition, the amplitude of the rhythm was nominally correlated with the timing of lunch (the preceding meal of siesta) toward a decreased amplitude with delayed timing (r = -0.648; *P* = 0.083). As compared with short nappers (≤30 min), long nappers had a significantly higher WHR and MetS score, whereas a trend was found for higher fasting glucose (nmol/L) (*P* = 0.054) and higher SBP (mmHg) (*P* = 0.068) (**Supplemental Table 1**). Concerning the characteristics of the *LIPE* rhythm, the amplitude was lower, the acrophase advanced, and the percent of rhythm lower in long-nappers than in short-nappers. However, no significant differences were reached (*P* > 0.1) (**Supplementary Table 1**).

## 4 Discussion

The current study was designed to obtain a better understanding of the molecular mechanisms that may underlie the link between napping and abdominal obesity. Data support our initial hypothesis and demonstrate that napping was associated with alterations in the pattern of the circadian expression of *LIPE*. Similar alterations were shown in the circadian expression of HSL activity, toward a robust circadian rhythm in non-nappers and a flattened rhythm among nappers. A lower amplitude and a lower percent of rhythm in *LIPE* rhythms were found in habitual nappers as compared with non-nappers. The decrease in amplitude in nappers was related to the frequency of siesta (times per week) toward lower amplitude with higher frequency. Previous studies have shown that the disruption of *LIPE* is related to a lower mobilization of lipids from adipose tissue (36), which may induce abdominal fat accumulation.

To our knowledge, this is the first study to analyze the circadian rhythm of *LIPE* expression taking into consideration the habit of napping. *LIPE*, encoding the enzyme hormone-sensitive lipase (HSL) (37), is mainly expressed in adipose tissue where it plays a regulatory role in lipolysis in adipocytes (38, 39). Our current results further suggest a similar alteration in the patterns of HSL activity among habitual nappers.

In line with our hypothesis, there are several studies investigating the effect of napping on abdominal obesity (40, 41) and metabolic health (3, 42, 43). Epidemiological and cross-sectional studies suggest that napping, especially long napping, is a risk factor for the development of cardiovascular diseases, diabetes (44–46), and obesity (5), especially abdominal obesity (43).

The results of the present work show that habitual nappers presented a significant flattening of the circadian rhythm of *LIPE* expression, with a 71% decrease in the amplitude of the rhythm as compared with non-nappers. Furthermore, similar results were found in HSL activity with a 21% decrease in the amplitude of the rhythm in nappers (as compared with non-nappers); nevertheless, significance was not reached. This decrease could be associated with greater difficulty in fat mobilization from adipose tissue depots. Flattening of the derived protein (HSL) rhythm has been associated with reduced fat mobilization in experimental animals (21). Further studies in HSL activity with a higher number of individuals should be performed to increase the statistical power of the sample.

In the total population studied, the acrophase of the *LIPE* rhythm was early at night (at 22:12 h), and the acrophase of HSL activity occurred just shortly afterward (at 23:41 h). These results are in agreement with previous studies revealing that lipolysis is maximal at night, potentially to provide sufficient energy for the night when the individual is usually sleeping and fasting (47). Furthermore, in habitual nappers, the percent of rhythm of *LIPE* decreased by 86% as compared with non-nappers. Similar results were found for HSL activity (a decrease of 35% in nappers as compared with non-nappers), although significance was not reached for the percent of rhythm in HSL activity.

Our results also show that the maximum expression of *LIPE*, which among non-nappers occurred at 20:00 h (in the timing point right after napping, CT12), was not present in nappers. This decrease in gene expression could be related to the habitual daytime sleep during napping in these subgroup of nappers since it resembles the decrease in *LIPE* expression that occurs at night right after the sleep onset in the subgroup of non-nappers. We hypothesize that an optimal circadian regulation of *LIPE* expression, with a high amplitude, and a peak at the beginning of the nighttime fasting episode (to support the extended overnight fast (48, 49)), would be associated with the appropriate distribution and mobilization of fat in adipose tissue, considering that the peak in HSL protein activity shortly follows the acrophase of the *LIPE* expression in 1.5 h (20). Results could help to explain why nappers are prone to abdominal obesity, which could be due to difficulties in abdominal fat mobilization.

While the biological mechanism that links siesta with abdominal obesity is still not clear, several interconnected reasons may explain the relationships between siesta, abdominal obesity, and metabolic syndrome (MetS) (40). Abdominal obesity may be due to disrupted circadian rhythms (50). Furthermore, the biological clock is known to play a fundamental role in regulating sleep and body weight (16, 51, 52) and the dysregulation in these rhythms has been associated with the development of MetS (9, 50, 53). Given that we are biologically designed to sleep at night (54), we could consider that daytime napping would be acting as a circadian disruptor. Previous studies have shown that cortisol, the circadian hormone *par* excellence, whose increase has been associated with abdominal obesity (55, 56), is elevated after awakening, not only in the morning after nighttime but also after daytime napping, which may partly explain the relationship between siesta, circadian system alterations, and obesity (10, 57).

The disruption of the circadian rhythm of *LIPE* with napping is demonstrated in our study, in which there is a total flattening of the rhythm, with a similar blunted rhythm in HSL activity. In addition, we observed that the frequency of napping per week was associated with this flattening, toward a lower amplitude among those who nap more frequently. The current study also shows a tendency toward a lower amplitude of the *LIPE* rhythm when eating a late lunch (the meal preceding siesta). Along this line, our recent epidemiological study performed in a Mediterranean area in Spain has shown that late eating of lunch is involved in the deleterious association between siesta and abdominal obesity (58).

This is the first study in proposing circadian rhythm alterations of a lipolytic gene (and of the activity of its protein) as a potential mechanism in the connection between napping and abdominal obesity. Although no significant differences between nappers and non-nappers were present in abdominal obesity traits, as the study was designed to match both groups, secondary analyses showed statistically significant differences between short nappers and long nappers for MetS score and its components, with increased values among long nappers. This is consistent with epidemiological and longitudinal studies that have shown an association between long siesta and abdominal obesity (41, 44, 58).

Among the strengths of the current study, this is the first study to observe a differential rhythmicity pattern of nappers from non-nappers in an *ex vivo* circadian pattern of *LIPE* in human adipose tissue. Results have been expanded with analysis of HSL activity. Furthermore, our study is unique because of the sample obtained of matched individuals in nappers and non-nappers, in a country where napping is a cultural habit.

Part of the limitations of the present work is that the study was performed in individuals with severe obesity and may not be directly transferable to the general population. We did not measure glycerol/FFA in the medium, which may be considered as a limitation. Furthermore, due to limitations in adipose tissue amount, we only determined data every 4 h during the 24 h of 1 day. While the collection of samples every 4 h has been widely used and accepted for circadian analysis, phase analyses may be uncertain. Furthermore, the siesta data were based on self-report. However, a previous study showed a relatively good concordance between objective and subjective measures of sleep-related parameters (59). In further studies, it is important to study whether napping is directly associated with decreased fat mobilization of body fat.

In summary, we have shown that *LIPE* expression in human AT shows a robust and marked endogenous rhythm in non-nappers, with maximal expression right before habitual sleep onset of the biopsy donors whereas in nappers the rhythm becomes flattened, results have been confirmed for HSL activity. These results may lead to a better insight into the underlying mechanisms involved in napping and abdominal obesity.

## Data availability statement

The datasets presented in this study can be found in online repositories. The names of the repository/repositories and accession number(s) can be found below: https://figshare.com/, https://doi.org/10.6084/m9.figshare.22093703.v2.

## Ethics statement

The studies involving human participants were reviewed and approved by Virgen de la Arrixaca University Hospital. The patients/participants provided their written informed consent to participate in this study.

## Author contributions

MG conceived and designed the study, acquired funding, designed the research, managed the project, and wrote the final version of the paper. FS conceived and designed the study and reviewed the final version of the manuscript. CZ contributed to the writing of the first draft of the manuscript, analyzed data, performed research, and designed all figures and tables. AK contributed to the analyses of *LIPE* expression and JL to the recruitment of patients and adipose tissue sampling. MR contributed analytic tools and intellectual advice and reviewed the paper. OM-A, FM, and BR-G performed protein analyses. All authors contributed to the manuscript revision and read and approved the submitted version.
