# Supplementary material for: Habitual nappers and non-nappers differ in circadian rhythms of LIPE expression in abdominal adipose tissue explants
Source: Front Endocrinol (Lausanne). 2023 Jun 9;14:1166961. doi: 10.3389/fendo.2023.1166961 (PMC10289256; doi:10.3389/fendo.2023.1166961)

Supplementary Material

Habitual nappers and non-nappers differ in circadian rhythms of *LIPE* expression in abdominal adipose tissue explants

Carolina Zambrano, Agné Kulyté, Juán Luján, Belén Rivero-Gutierrez, Fermín Sanchez de Medina, Olga Martínez-Augustin, Mikael Ryden, Frank A. J. L. Scheer, Marta Garaulet.

* Correspondence: Marta Garaulet

# Supplementary Data

Raw data Gen *LIPE* expression

|  |  | **Sybr** |  |  | **Sybr** |
| --- | --- | --- | --- | --- | --- |
| **Gene** | **Number Patients-circadian timing** | **cT values** | **Gene** | **Number Patients-circadian timing** | **cT values** |
| 18s_Sybr | P2_0 | 15.66 | LIPE_Sybr | P2_0 | 29.43 |
| 18s_Sybr | P3_0 | 15.84 | LIPE_Sybr | P3_0 | 29.55 |
| 18s_Sybr | P4_0 | 14.03 | LIPE_Sybr | P4_0 | 27.64 |
| 18s_Sybr | P5_0 | 18.51 | LIPE_Sybr | P5_0 | 31.57 |
| 18s_Sybr | P6_0 | 14.32 | LIPE_Sybr | P6_0 | N/A |
| 18s_Sybr | P7_0 | 15.94 | LIPE_Sybr | P7_0 | 29.45 |
| 18s_Sybr | P8_0 | 19.16 | LIPE_Sybr | P8_0 | 31.54 |
| 18s_Sybr | p9_0 | 14.05 | LIPE_Sybr | p9_0 | 25.31 |
| 18s_Sybr | P10_0 | 16.42 | LIPE_Sybr | P10_0 | N/A |
| 18s_Sybr | p11_0 | 14.91 | LIPE_Sybr | p11_0 | 27.07 |
| 18s_Sybr | p12_0 | 15.41 | LIPE_Sybr | p12_0 | 28.56 |
| 18s_Sybr | p13_0 | 16.21 | LIPE_Sybr | p13_0 | 29.95 |
| 18s_Sybr | p14_0 | 14.44 | LIPE_Sybr | p14_0 | 27.19 |
| 18s_Sybr | p15_0 | 14.35 | LIPE_Sybr | p15_0 | 26.44 |
| 18s_Sybr | p16_0 | 14.66 | LIPE_Sybr | p16_0 | 27.42 |
| 18s_Sybr | p17_0 | 13.84 | LIPE_Sybr | p17_0 | 24.99 |
| 18s_Sybr | p18_0 | 17.39 | LIPE_Sybr | p18_0 | 29.85 |
| 18s_Sybr | P2_4 | 14.15 | LIPE_Sybr | P2_4 | 37.16 |
| 18s_Sybr | P3_4 | 15.4 | LIPE_Sybr | P3_4 | 29.12 |
| 18s_Sybr | P4_4 | 15.22 | LIPE_Sybr | P4_4 | 28.76 |
| 18s_Sybr | P5_4 | 18.9 | LIPE_Sybr | P5_4 | 31.55 |
| 18s_Sybr | P6_4 | 14.16 | LIPE_Sybr | P6_4 | 36.6 |
| 18s_Sybr | P7_4 | 16.18 | LIPE_Sybr | P7_4 | 30.62 |
| 18s_Sybr | P8_4 | 17.18 | LIPE_Sybr | P8_4 | 31.12 |
| 18s_Sybr | P9_4 | 15.12 | LIPE_Sybr | P9_4 | 25.57 |
| 18s_Sybr | P10_4 | 15.32 | LIPE_Sybr | P10_4 | 36.76 |
| 18s_Sybr | p11_4 | 14.39 | LIPE_Sybr | p11_4 | 25.50 |
| 18s_Sybr | p12_4 | 15.21 | LIPE_Sybr | p12_4 | 28.14 |
| 18s_Sybr | p13_4 | 14.56 | LIPE_Sybr | p13_4 | 27.09 |
| 18s_Sybr | p14_4 | 14.73 | LIPE_Sybr | p14_4 | 26.49 |
| 18s_Sybr | p15_4 | 15.31 | LIPE_Sybr | p15_4 | 27.07 |
| 18s_Sybr | p16_4 | 15.2 | LIPE_Sybr | p16_4 | 27.76 |
| 18s_Sybr | p17_4 | 14.4 | LIPE_Sybr | p17_4 | 25.27 |
| 18s_Sybr | p18_4 | 15.49 | LIPE_Sybr | p18_4 | 26.92 |
| 18s_Sybr | P2_8 | 15.54 | LIPE_Sybr | P2_8 | 29.42 |
| 18s_Sybr | P3_8 | 14.75 | LIPE_Sybr | P3_8 | 27.16 |
| 18s_Sybr | P4_8 | 16.08 | LIPE_Sybr | P4_8 | 28.77 |
| 18s_Sybr | P5_8 | 16.86 | LIPE_Sybr | P5_8 | 30.13 |
| 18s_Sybr | P6_8 | 13.99 | LIPE_Sybr | P6_8 | 26.58 |
| 18s_Sybr | P7_8 | 16.12 | LIPE_Sybr | P7_8 | 29.42 |
| 18s_Sybr | P8_8 | 15.06 | LIPE_Sybr | P8_8 | 28.49 |
| 18s_Sybr | P9_8 | 14.49 | LIPE_Sybr | P9_8 | 24.82 |
| 18s_Sybr | P10_8 | 14.59 | LIPE_Sybr | P10_8 | 28.02 |
| 18s_Sybr | p11_8 | 14.77 | LIPE_Sybr | p11_8 | 25.94 |
| 18s_Sybr | p12_8 | 16.08 | LIPE_Sybr | p12_8 | 28.03 |
| 18s_Sybr | p13_8 | 14.99 | LIPE_Sybr | p13_8 | 28.28 |
| 18s_Sybr | p14_8 | 14.78 | LIPE_Sybr | p14_8 | 26.70 |
| 18s_Sybr | p15_8 | 15.45 | LIPE_Sybr | p15_8 | 28.11 |
| 18s_Sybr | p16_8 | 14.97 | LIPE_Sybr | p16_8 | 26.07 |
| 18s_Sybr | p17_8 | 15.51 | LIPE_Sybr | p17_8 | 26.98 |
| 18s_Sybr | p18_8 | 14.79 | LIPE_Sybr | p18_8 | 25.41 |
| 18s_Sybr | P2_12 | 13.11 | LIPE_Sybr | P2_12 | 28.48 |
| 18s_Sybr | P3_12 | 15.33 | LIPE_Sybr | P3_12 | 28.5 |
| 18s_Sybr | P4_12 | 16 | LIPE_Sybr | P4_12 | 29.07 |
| 18s_Sybr | P5_12 | 17.17 | LIPE_Sybr | P5_12 | 36.56 |
| 18s_Sybr | P6_12 | 14.42 | LIPE_Sybr | P6_12 | 26.19 |
| 18s_Sybr | P7_12 | 15.98 | LIPE_Sybr | P7_12 | 28.61 |
| 18s_Sybr | P8_12 | 16.04 | LIPE_Sybr | P8_12 | 28.63 |
| 18s_Sybr | P9_12 | 15.39 | LIPE_Sybr | P9_12 | 25.65 |
| 18s_Sybr | P10_12 | 15.67 | LIPE_Sybr | P10_12 | 29.03 |
| 18s_Sybr | p11_12 | 15.65 | LIPE_Sybr | p11_12 | 26.16 |
| 18s_Sybr | p12_12 | 15.84 | LIPE_Sybr | p12_12 | 27.52 |
| 18s_Sybr | p13_12 | 15.83 | LIPE_Sybr | p13_12 | 27.84 |
| 18s_Sybr | p14_12 | 15.13 | LIPE_Sybr | p14_12 | 27.09 |
| 18s_Sybr | p15_12 | 16.21 | LIPE_Sybr | p15_12 | 29.26 |
| 18s_Sybr | p16_12 | 15.55 | LIPE_Sybr | p16_12 | 28.18 |
| 18s_Sybr | p17_12 | 15.14 | LIPE_Sybr | p17_12 | 25.77 |
| 18s_Sybr | p18_12 | 17.33 | LIPE_Sybr | p18_12 | 26.13 |
| 18s_Sybr | P2_16 | 15.12 | LIPE_Sybr | P2_16 | 29.02 |
| 18s_Sybr | P3_16 | 15.36 | LIPE_Sybr | P3_16 | 28.7 |
| 18s_Sybr | P4_16 | 15.66 | LIPE_Sybr | P4_16 | 29.38 |
| 18s_Sybr | P5_16 | 17.28 | LIPE_Sybr | P5_16 | 29.81 |
| 18s_Sybr | P6_16 | 14.29 | LIPE_Sybr | P6_16 | 27.31 |
| 18s_Sybr | P7_16 | 16.5 | LIPE_Sybr | P7_16 | 30.43 |
| 18s_Sybr | P8_16 | 14.84 | LIPE_Sybr | P8_16 | 28.06 |
| 18s_Sybr | P9_16 | 15.11 | LIPE_Sybr | P9_16 | 25.57 |
| 18s_Sybr | P10_16 | 14.12 | LIPE_Sybr | P10_16 | 26.56 |
| 18s_Sybr | p11_16 | 15.45 | LIPE_Sybr | p11_16 | 25.97 |
| 18s_Sybr | p12_16 | 15.88 | LIPE_Sybr | p12_16 | 27.77 |
| 18s_Sybr | p13_16 | 14.75 | LIPE_Sybr | p13_16 | 27.08 |
| 18s_Sybr | p14_16 | 14.56 | LIPE_Sybr | p14_16 | 26.36 |
| 18s_Sybr | p15_16 | 15.01 | LIPE_Sybr | p15_16 | 26.37 |
| 18s_Sybr | p16_16 | 15.01 | LIPE_Sybr | p16_16 | 25.22 |
| 18s_Sybr | p17_16 | 14.95 | LIPE_Sybr | p17_16 | 25.81 |
| 18s_Sybr | p18_16 | 16 | LIPE_Sybr | p18_16 | 26.27 |
| 18s_Sybr | P2_20 | 15.77 | LIPE_Sybr | P2_20 | 29.7 |
| 18s_Sybr | P3_20 | 15.02 | LIPE_Sybr | P3_20 | 28.47 |
| 18s_Sybr | P4_20 | 15.66 | LIPE_Sybr | P4_20 | 28.58 |
| 18s_Sybr | P5_20 | 14.52 | LIPE_Sybr | P5_20 | 27.76 |
| 18s_Sybr | P6_20 | 14.57 | LIPE_Sybr | P6_20 | 27.33 |
| 18s_Sybr | P7_20 | 18.19 | LIPE_Sybr | P7_20 | 32.65 |
| 18s_Sybr | P8_20 | 14.86 | LIPE_Sybr | P8_20 | 29.25 |
| 18s_Sybr | P9_20 | 14.86 | LIPE_Sybr | P9_20 | 25.34 |
| 18s_Sybr | P10_20 | 15.93 | LIPE_Sybr | P10_20 | 29.26 |
| 18s_Sybr | p11_20 | 15.57 | LIPE_Sybr | p11_20 | 25.88 |
| 18s_Sybr | p12_20 | 15.17 | LIPE_Sybr | p12_20 | 27.77 |
| 18s_Sybr | p13_20 | 15.23 | LIPE_Sybr | p13_20 | 28.33 |
| 18s_Sybr | p14_20 | 14.55 | LIPE_Sybr | p14_20 | 26.32 |
| 18s_Sybr | p15_20 | 15.2 | LIPE_Sybr | p15_20 | 28.14 |
| 18s_Sybr | p16_20 | 15.01 | LIPE_Sybr | p16_20 | 25.63 |
| 18s_Sybr | p17_20 | 15.08 | LIPE_Sybr | p17_20 | 26.07 |
| 18s_Sybr | p18_20 | 15.15 | LIPE_Sybr | p18_20 | 25.72 |

# Supplementary Table

**Supplementary Table 1.** Characteristics of the metabolic syndrome traits and 24h circadian rhythms of *LIPE* expression of the participants. in short and long duration nap.

|  | **Categories of Daytime Napping (min)** | | | |  |
| --- | --- | --- | --- | --- | --- |
|  | **≤30 min**  **n=3** | | **> 30 min**  **n=5** | | **Student T test** |
|  | Mean | SD | Mean | SD | p-value |
| **CHARACTERISTICS** |  |  |  |  |  |
| Metabolic Syndrome Traits |  |  |  |  |  |
| WHR | 0.87 | 0.01 | 1.03 | 0.09 | 0.029 |
| *Glucose (nmol/L) | 5.19 | 0.13 | 8.34 | 2.20 | 0.054 |
| Systolic BP (mmHg) | 128.6 | 3.51 | 143.2 | 10.68 | 0.068 |
| Diastolic BP (mmHg) | 80.3 | 9.07 | 85.60 | 7.19 | 0.395 |
| MetS | 2.00 | 1.73 | 4.20 | 0.44 | 0.030 |
| **Rhythm characteristics LIPE expression** |  |  |  |  |  |
| Average (fold change) | 12.62 | 1.83 | 13.29 | 1.46 | 0.583 |
| Amplitude (% normalized) | 0.31 | 0.09 | 0.29 | 0.17 | 0.818 |
| Acrophase (hh:mm) | 23:52 | 7:31 | 23:15 | 4:21 | 0.931 |
| Percent of rhythm | 45.73 | 20.84 | 28.85 | 19.99 | 0.298 |

WHR. waist-hip ratio**;** BP. blood pressure; MetS. Metabolic syndrome; *Fasting conditions.

**Supplementary Table 2.** Characteristics of 24h circadian rhythms of Hormone-sensible Lipase activity in nappers and non-nappers.

|  | **Total Population (n=17)** | | **Napper**  **(n=8)** | | **Non-napper**  **(n=9)** | | **ANCOVA** | **ANCOVA** |
| --- | --- | --- | --- | --- | --- | --- | --- | --- |
|  | Mean | SD | Mean | SD | Mean | SD | p-values* | p-values** |
| **Rhythm characteristics HSL activity expression** |  |  |  |  |  |  |  |  |
| Average (fold change or ΔCt ) | 0.77 | 0.42 | 0.79 | 0.55 | 0.75 | 0.30 | 0.895 | 0.841 |
| Amplitude (% normalized) | 0.18 | 0.12 | 0.16 | 0.13 | 0.20 | 0.10 | 0.555 | 0.564 |
| Acrophase (hh:mm) | 23:41 | 5:58 | 00:49 | 5:04 | 19:41 | 4:09 | 0.100 | 0.056 |
| Percent of rhythm | 38.78 | 26.47 | 30.26 | 26.29 | 46.35 | 25.68 | 0.211 | 0.170 |
|  |  |  | **Significance** | | **Significance** | |  |  |
| Circadian rhythmicity (*P* value) |  |  | 0.112 | | 0.020 | |  |  |

*Adjusted for sex and age **Adjusted for sex, age, and sleep duration night. Data were obtained from the median of each individual´s rhythm characteristics.

# Supplementary Figure

**Figure 1**. HSL signaling in adipose tissue. Phosphorylated HSL (pHSL) and total HSL (tHSL) were measured by Western blot in cultured tissue samples obtained every 4 hours for 24 hours at the following times (CT0 wake time/medium change [08:00] CT4 [12:00 h], ZT8 [16:00 h], CT12 [20:00 h], CT16 [00:00 h], and CT20 [04:00 h]). Panel A and B show an example of the Western blot performed for pHSL and tHSL in one individual (napper) and the corresponding levels of quantification. Panel C and Panel D show a similar representation but for one non-napper.

#
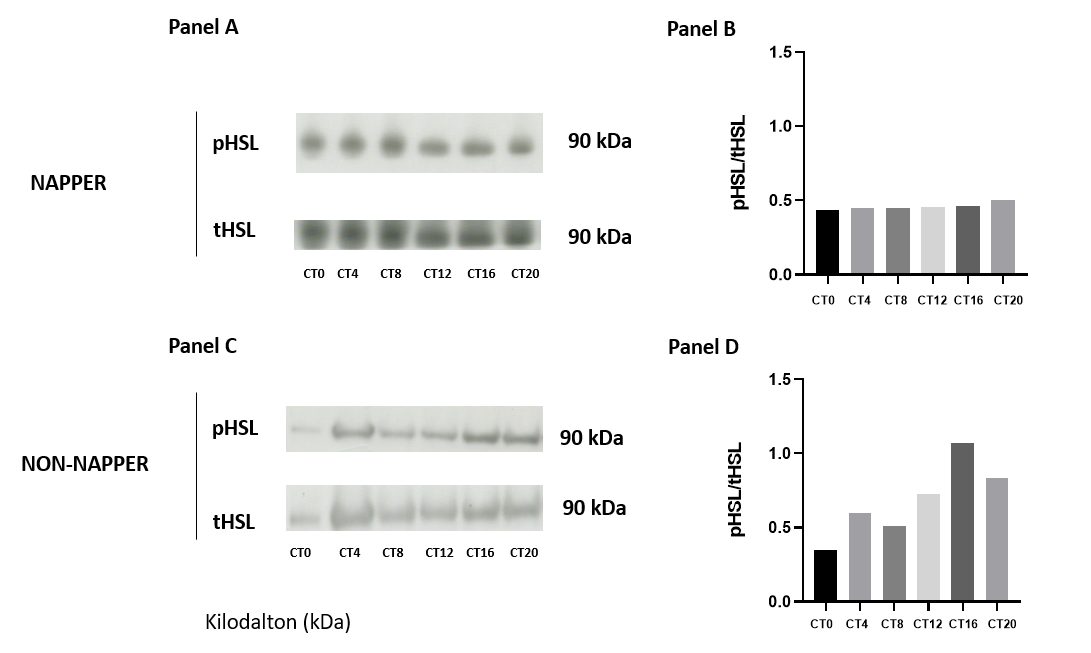

Supplement: Supplementary file 1 [file DataSheet_1.doc]
